# Implementation of Chaotic Reverse Slime Mould Algorithm Based on the Dandelion Optimizer

**DOI:** 10.3390/biomimetics8060482

**Published:** 2023-10-11

**Authors:** Yi Zhang, Yang Liu, Yue Zhao, Xu Wang

**Affiliations:** College of Electrical and Computer Science, Jilin Jianzhu University, Changchun 130119, China; 18695888056@163.com (Y.L.); zhaoyue@jlju.edu.cn (Y.Z.); wangxu@jlju.edu.cn (X.W.)

**Keywords:** Bernoulli chaotic mapping, Lévy flight, short-term load forecasting, slime mould algorithm

## Abstract

This paper presents a hybrid algorithm based on the slime mould algorithm (SMA) and the mixed dandelion optimizer. The hybrid algorithm improves the convergence speed and prevents the algorithm from falling into the local optimal. (1) The Bernoulli chaotic mapping is added in the initialization phase to enrich the population diversity. (2) The Brownian motion and Lévy flight strategy are added to further enhance the global search ability and local exploitation performance of the slime mould. (3) The specular reflection learning is added in the late iteration to improve the population search ability and avoid falling into local optimality. The experimental results show that the convergence speed and precision of the improved algorithm are improved in the standard test functions. At last, this paper optimizes the parameters of the Extreme Learning Machine (ELM) model with the improved method and applies it to the power load forecasting problem. The effectiveness of the improved method in solving practical engineering problems is further verified.

## 1. Introduction

A meta-heuristic algorithm [1] is an algorithm based on stochastic operators that does not depend on gradient information. It finds better solutions with limited computing power and is suitable for solving complex problems with continuous, discrete or even mixed search spaces. It has been widely used in solving practical engineering problems because of its simple concept and easy implementation. These algorithms generally fall into four categories: swarms’ behavior-based, physical rule-based, nature-based, and human-related algorithms. Swarms with collective behavior inspire swarming algorithms. The most famous are the Particle Swarm Optimization (PSO) [2] and the Ant Colony Optimization (ACO) [3]. The Whale Optimization Algorithm (WOA) [4], the Marine Predator Algorithm (MPA) [5], the Artificial Gorilla Troops Optimizer (GTO) [6], the Snake swarm Optimizer (SO) [7], and the Nutcracker Optimization Algorithm (NOA) [8] have also been proposed in recent years. Physical laws and mathematical rules mostly inspire physics-based algorithms. Such algorithms usually have strict proofs. The typed algorithms are the Simulated Annealing (SA) [9], the Multi-verse Optimizer (MO) [10], the Sine–Cosine Algorithm (SCA) [11], and the Kepler Optimization Algorithm (KOA) [12]. Nature-based algorithms are primarily derived from biological evolution in nature, such as the Genetic Algorithm (GA) [13], the Differential Evolution algorithm (DE) [14], and the Evolutionary Strategy (ES) [15]. Human-related algorithms are developed from long-term human experiences, such as the harmony algorithm [16], the Teaching-Based Optimization (TLBO) [17], and the League Championship algorithm [18]. Exploration and exploitation are the two most essential parts of the meta-heuristic process. The exploration phase refers to searching the solution space as broadly, randomly, and globally as possible. The exploitation stage refers to the ability of the algorithm to search more accurately in the area acquired in the exploration stage, with reduced randomness and improved accuracy [19]. However, over-exploration will eventually lead to convergence difficulties, and only focusing on exploitation will cause the model to easily fall into local optimization. Therefore, how to strike a balance between exploration and utilization is a complex problem for meta-heuristic algorithms.

The optimization algorithm selected in this paper is the slime mould algorithm (SMA) proposed by Li et al. in 2020 [20]. The SMA is inspired by slime molds’ behavior and morphological changes during foraging. The SMA has been applied to various engineering optimization problems because of its simple code and few parameters. However, SMA needs to improve in dealing with complex and high-dimensional problems. Researchers have continuously optimized the SMA in recent years. There are generally two kinds of improved strategies: one is to improve the core equation of the algorithm by using a variety of strategies, and the other is to mix a variety of algorithms to improve efficiency. Yu et al. [21] combined reverse learning and chaotic mapping to optimize SMA and performed well in urban water resources treatment. Naik et al. [22] proposed to add adaptive reverse learning at the later stage of iteration to avoid the premature end of convergence. Zhang et al. [23] presented reverse learning and Quantum Rotation Gate strategies to the SMA. Jiang et al. [24] proposed an improved SMA based on elite reverse learning. The adaptive probability threshold was adopted to adjust the selection probability of slime moulds. The quality and diversity of the initial population are improved. Alfadhli et al. [25] chose to integrate adaptive parameters into the iteration of the population. The improved method adaptively changes the population size to effectively balance the characteristics of exploitation and exploration in different stages of the SMA. Liu et al. [26] introduced Chebyshev chaotic mapping in the initialization stage. They added the simplex method in the later exploration stage to increase local search ability and avoid premature convergence, which achieved excellent results in extracting PV model parameters. Qiu et al. [27] proposed a mechanism for updating locations in stages, which divided the iteration time into three segments on average. Different stages mix different optimization strategies to balance exploitation and exploration; researchers have also integrated other swarm intelligence algorithms with SMA [28,29,30,31,32], respectively, in either the exploration or exploitation stage to carry out different degrees of optimization, and achieved excellent results in image segmentation, support vector regression (SVR) prediction problems, and other directions.

These algorithm improvements perform well in their respective domains. However, the performance improvements on one class of problems will be offset by performance declines on another class of problems according to the No free Lunch (NFL) theorem [33], and any other algorithm will not be ideally suited to handle various problems. Therefore, it is necessary to improve the corresponding algorithm according to the different requirements of the problem. 

This paper presents an improved slime mould algorithm to solve the problems related to the actual power load prediction accuracy and stability: (1) The Bernoulli chaotic mapping is added in the initial stage because the proportion of new individuals randomly generated in the initial stage is tiny, resulting in insufficient randomness. Moreover, the initial population is optimized by using the randomness and ergodicity of the chaotic mapping. It makes the distribution of slime moulds more reasonable and avoids premature puberty. (2) The decision parameter p is evenly divided into two stages, and then the excellent mechanism of development is explored in different stages in the mixed DO algorithm. The algorithm adopts different location update formulas at different stages to increase the diversity of the distribution of slime moulds and further enhance the global search ability and local exploitation. (3) The specular reflection learning strategy is introduced in the late iteration to help the group escape from local optimization and improve the solution accuracy. 

The rest of this article is structured as follows: Section 2 describes the principal concepts of the SMA and the DO. Section 3 introduces the details of the improved algorithm BDSSMA and the improved mathematical model. In Section 4, the proposed algorithm is compared with six swarm intelligence algorithms based on 23 benchmark functions to evaluate the performance of the proposed algorithm, and the statistical validity of the proposed algorithm is evaluated via the Wilcoxon rank sum test. In Section 5, the power load forecasting model of ELM is used to test the above several population intelligent algorithms in practical engineering problems and prove their feasibility in power load forecasting problems. Section 6 summarizes the whole work and provides some inspirations for future work.

## 2. Background

### 2.1. Slime Mould Algorithm (SMA)

The SMA is a population intelligent algorithm based on slime molds’ behavior and morphological changes in foraging. Its foraging behavior is mainly divided into two stages. The corresponding mathematical model and method will be briefly summarized in the following sections.

#### 2.1.1. Approaching the Food Stage

Slime moulds will approach food according to the concentration of odor in the air, and this contraction pattern of approaching food can be defined as:

(1) $$X(t+1)=\left\{\begin{array}{c} X_{b}(t)+vb\cdot (W\cdot X_{A}(t)-X_{B}(t)),r<p\\ vc\cdot X(t),r\geq p \end{array}\right.$$

In the formula, $X_{b}(t)$ represents the position of the individual with the highest food concentration found so far, that is, the current global optimal solution; $X_{A}\left(t\right)$ and $\;X_{B}\left(t\right)\;$ represent two individuals randomly selected from the population; X(t) represents the position of slime mould; W represents the weight of slime mould individuals. vb is a vibration parameter randomly valued in the interval  [−a, a]; vc  is a random value linearly decreasing from 1 to 0; t represents the number of current iterations; *r* represents the random value within the interval [0, 1]; and the mathematical model description of the control variable p and the range parameter of the disturbance interval a  is described as:

(2) $$p=\tanh\left|S(i)-bF\right|$$

(3) $$a=arctanh(-\left(\frac{t}{t_{max}}\right)+1)$$

where, i ∈ 1, 2, 3…n, S(i) represents the fitness value of the current individual X(t), bF is the current best fitness value, and $t_{max}$ is the maximum number of iterations. The mathematical model description of the weight parameter W is shown in Equations (4) and (5):

(4) $$W\left(SmellIndex\left(i\right)\right)=\left\{\begin{array}{c} 1+r\cdot \log(\frac{bF-S(i)}{bF-\omega F}+1),condition\\ 1-r\cdot \log(\frac{bF-S(i)}{bF-\omega F}+1),others \end{array}\right.$$

(5) SmellIndex(i)=Sort(S)

In the formula, the condition indicates that S(i) sorts the first half of the population, bF represents the optimal fitness obtained during the current iteration, ωF represents the worst fitness value obtained during the current iteration, and *log* is used to reduce the change rate of the value, so that the contraction frequency will not change too much. SmellIndex represents the sequence of fitness values for the sort (ascending order is used in minimum problems).

#### 2.1.2. Stage of Wrapping Food

In this stage, the constriction pattern of a vein tissue structure was simulated during the search for moulds. The higher the concentration of food in venous contact, the stronger the waves produced by the biological oscillator, the faster the cytoplasmic flow, and the thicker the veins [20]. Equation (4): the positive and negative feedback between the vein width of the slime mould and the food concentration being explored is simulated to adjust its search pattern according to the food quality. When the food concentration is the content, the weight near the region is more significant; when the food concentration is low, the area’s importance is reduced, and the exploration of the other regions is shifted. Based on the above principles, the mathematical formula of Equation (6) for slime mould location is updated as follows:

(6) $$X\left(t+1\right)=\left\{\begin{array}{c} rand\cdot \left(UB-LB\right)+LB,rand<Z\\ X_{b}\left(t\right)+vb\cdot \left(W\cdot X_{A}\left(t\right)-X_{B}\left(t\right)\right),rand\geq Z,r<p\\ vc\cdot X(t),r\geq p \end{array}\right.$$

where *UB* and *LB* represent the upper and lower boundaries of the search range, *rand* and *r* represent random values in [0, 1]. Z is a parameter used to balance exploration and exploitation. In [20], Li proved through many experiments that Z of 0.03 is the best result.

### 2.2. Dandelion Optimizer

The dandelion optimizer (DO) is a novel swarm intelligence algorithm proposed by Zhao et al [1], in 2022, to simulate the behavior of dandelion seeds in long-distance flight, relying on wind. The process is mainly divided into three stages [1].

#### 2.2.1. Ascending Phase

In the ascending phase, it is usually divided into two conditions: sunny or rainy days.

##### Situation 1: Sunny Day

On a clear day, the wind speed can be viewed as having a logarithmic normal distribution $ln\;Y\;\sim \;N(\mu ,\sigma^{2})$. Under this distribution, the random numbers are distributed more along the 𝑌 axis, which allows the dandelion seeds to spread further. In this case, DO emphasizes exploration, in which the dandelion seeds are randomly blown by the wind to various locations in the search space. The height of the dandelion seeds is determined by the wind speed. The higher the wind, the higher the dandelion seeds fly and the farther the seeds scatter. Affected by the wind speed, the vortex above the dandelion seeds is constantly adjusted to make it rise in a spiral. In this case, the corresponding mathematical expression is:

(7) $$X_{t+1}=X_{t}+\alpha \cdot v_{x}\cdot v_{y}\cdot lnY\cdot (X_{s}-X_{t})$$

lnY denotes a lognormal distribution subject to 𝜇 = 0 and $\sigma^{2}$ = 1, and its mathematical formula is:

(8) $$lnY=\left\{\begin{array}{cc} \frac{1}{y\sqrt{2\;\pi }}exp\left[-\frac{1}{2\sigma^{2}}{\left(lny\right)}^{2}\right] & y\geq 0\\ 0 & y<0 \end{array}\right.$$

where $X_{t}$ represents the position of the dandelion seed in t iterations. $X_{s}$ represents a randomly selected position in the search space over t iterations. y denotes the standard normal distribution 𝑁 (0, 1). The expression of randomly generated positions is shown in Equation (9):

(9) $$\alpha =rand()\cdot \left(\frac{t^{2}}{{t_{max}}^{2}}-\frac{2t}{t_{max}}+1\right)$$

where α is a linearly decreasing random value from 1 to 0. θ is a random number between [−𝜋, 𝜋]. Such fluctuations make the algorithm pay much attention to global search in the early stage and turn to local search in the later stage, which is conducive to ensuring accurate convergence after global search. $v_{x}$ and $v_{y}$ represent the lift component coefficients of dandelion due to the separation vortex action, and r is used as the variable with more randomness. Equation (10) is the corresponding mathematical expression:

(10) $$\left\{\begin{array}{c} r=\frac{1}{e^{\theta }}\\ v_{x}=r\cdot cos\theta \\ v_{y}=r\cdot sin\theta \;\;\;\;\; \end{array}\right.$$

##### Situation 2: Rainy Days

On rainy days, dandelion seeds cannot rise properly with the wind due to factors such as air resistance and humidity. In this case, dandelion seeds are developed locally, and the corresponding mathematical expression is:

(11) $$X_{t+1}=X_{t}\cdot k$$

where k is used to adjust the local search area of the dandelion (Equation (12)), and Equation (13) is used to calculate the domain:

(12) $$q=1+\frac{{\left(t-1\right)}^{2}}{{\left(T-1\right)}^{2}}$$

(13) k=1−rand()⋅ q

#### 2.2.2. Decline Stage

At this stage, dandelion seeds are still mainly explored. It is when the dandelion seed rises to a certain distance after a steady decline. Because Brownian motion follows normal distribution in each change, it is easy for individuals to traverse more search areas in the iterative updating process. Therefore, Brownian motion is selected to simulate the motion trajectory of dandelion seeds in the descending process. In order to reflect the stability of dandelion seeds in the descending stage, the average position information after the ascending stage is adopted. This promotes the exploitation of the whole population into better areas. The corresponding mathematical expression is:

(14) $$X_{t+1}=X_{t}-\alpha \cdot \beta_{t}\cdot (X_{mean-t}-\alpha \cdot \beta_{t}\cdot X_{t})$$

(15) $$X_{mean-t}=\frac{1}{pop}\sum_{i=1}^{pop}X_{i}$$

In the formula, $\beta_{t}$ represents the Brownian motion and is a random number with standard normal distribution. $X_{mean-t}$ represents the average position of the population in the iteration.

#### 2.2.3. Landing Phase

In this section, dandelion seeds are turned into exploitation. With the continuous iteration in the first two stages, it is possible for the algorithm to converge to the global optimal solution at present. Therefore, the optimal solution obtained at present is the approximate location where dandelion seeds are most likely to survive. In order to accurately converge to the global optimal, the search individual selects the current optimal solution for exploitation in the current region. With the continuous evolution of the population, the global optimal solution can finally be found, and the corresponding mathematical expression is:

(16) $$X_{t+1}=X_{elite}+levy(\lambda )\cdot \alpha \cdot (X_{elite}-X_{t}\cdot \delta )$$

where $X_{elite}$ represents the best position of the dandelion seed in the i iteration. levy (λ) represents the Lévy flight function, *𝛿* is a linearly increasing function from 0 to 2, and the corresponding mathematical expression is:

(17) $$levy\left(\;\lambda \right)=s\cdot \frac{\omega \times \sigma }{{\left|t\right|}^{\frac{1}{\beta }}}$$

(18) $$\delta =\frac{2t}{T}$$

where β is a random number between [0, 2] (β = 1.5 in this article). s is a fixed constant of 0.01. Both ω and t are random numbers between [0, 1]. The mathematical expression of σ is:

(19) $$\sigma =\left(\frac{\Gamma (1+\beta ))\cdot sin(\frac{\pi \beta }{2})}{\Gamma \left(\frac{1+\beta }{2}\right)\cdot \beta \times 2^{\left(\frac{\beta -1}{2}\right)}}\right)$$

## 3. Methods

In the second part, we find that SMA is an algorithm with simple parameters, stable operation, and particular optimization ability. However, there are still some problems: First, the initial population of the swarm intelligence algorithm should have diversity, but the random parameter *Z* of SMA is only 0.03, which is a small constant. The proportion of new individuals randomly generated by Equation (6) in the total population is tiny, and the population diversity will also decrease with the update of individual positions, resulting in the local optimization of the algorithm. It could perform better at jumping out and reexploring. Secondly, from the perspective of the slime mould position update mechanism, Equation (1), the position update of the slime mould is determined by the position of the current optimal individual and the position of two random individuals, which is equivalent to random exploration near the current optimal position. This enhances the global search ability of SMA in the early stage to some extent, but two randomly selected individuals also slow down the convergence rate of SMA. As the iteration progresses, the population tends to move closer to the current optimal position, which makes it easy for SMA to fall into local optima when solving functions with multiple local optima. Finally, in the exploitation stage, the disturbance factor vc converges linearly from 1 to 0. This simple linear function is easy to make the slime mould individual start slowly in the later exploitation, resulting in slow algorithm convergence speed or insufficient solution accuracy.

This paper proposed the following changes to solve the above problem: First, Bernoulli chaotic mapping was added in the initialization stage, and the randomness and ergodicity of the chaotic mapping were used to optimize the initial population to make the individual distribution of slime moulds more reasonable and avoid premature puberty. Second, the control variable p is divided into two stages, and then the excellent mechanism of stage exploration and exploitation is mixed in DO to increase the diversity of molds’ individual distribution, so that the algorithm adopts different position update formulas at various stages, and further enhances the global search ability and local exploitation performance of moulds. Thirdly, the planar mirror reflection imaging learning strategy is introduced in the late iteration to help the group escape from local optimization and improve the solution accuracy. The improvement measures are described as follows.

### 3.1. Chaotic Mapping

Whether the population initialization is uniform is an essential factor in determining the optimization effect of the algorithm. Therefore, chaotic mapping is introduced to initialize the algorithm population, which can improve the initial population’s diversity and improve the population’s quality in subsequent iterations. In ref. [26], Liu et al. concluded that Chebyshev chaos mapping has the best optimization effect on the initialization stage of SMA compared with 10 common chaos factors. However, in addition to the mentioned chaos factors, other outstanding chaos factors have yet to be discussed. We compare the other chaos factors [34] (Table 1) with the best chaos map currently available in SMA (Chebyshev’s chaos map) and discuss whether there are better alternatives.

### 3.2. Optimization of Location Update Mechanism

As mentioned above, researchers mainly deal with the main impact factors in stages for the optimization of SMA position update mechanism, such as the average number of iterations *t* and weight coefficient *ω* into multiple stages, and different stages integrate different strategy mechanisms to achieve the optimization and balance of exploration and exploitation. However, no researchers have optimized the position update decision parameter *p*. In this paper, it is proposed for the first time that parameter *p* is evenly divided into two segments, and the different mechanisms that dandelion seeds rely on in different landing stages in the DO are mixed, such as the Lévy flight strategy and Brownian motion. The following section describes how these two mechanisms improve the location update section.

**Figure 1:**
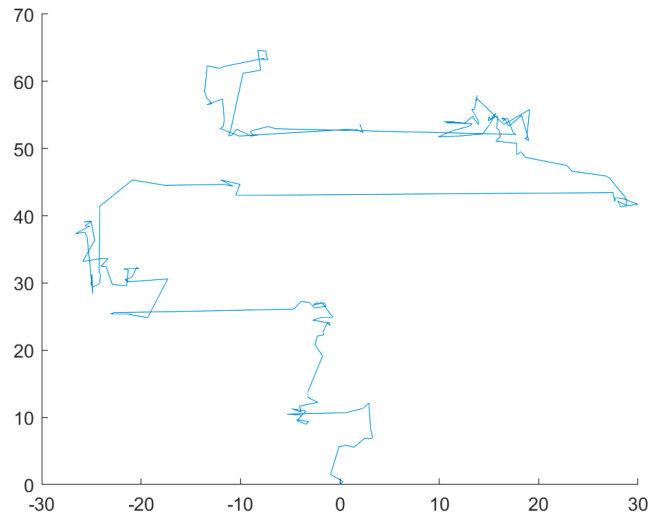
Lévy flight.

**Figure 2:**
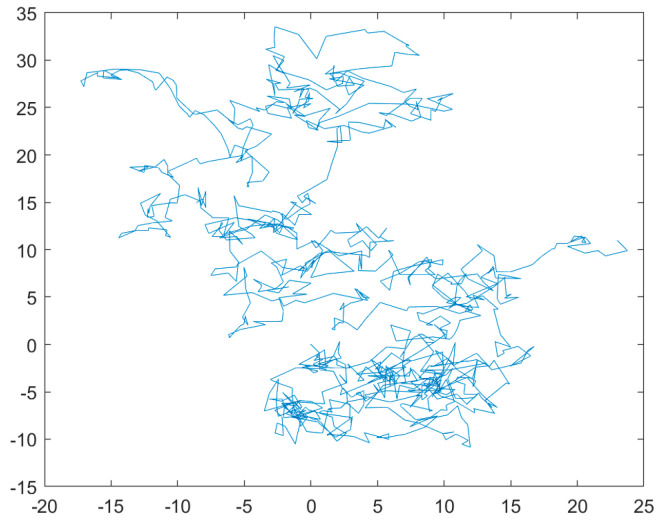
Brownian movement.

First, according to the two-dimensional trajectory diagram of Lévy’s flight strategy and Brownian motion in Figure 1 and Figure 2, Lévy’s flight trajectory has irregular step size, small and uncertain step size, and a larger search area. In contrast, Brownian motion has a more uniform and controlled step size, allowing for a better coverage of the entire area for finer exploitation.

Therefore, Brownian motion is added in the pre-$\frac{p}{2}$ part at the stage of lower food concentration. In the original SMA, two random individuals are used to search at this stage. Although the randomly selected individuals can increase the search scope to some extent, they will lead to a slower convergence of SMA. This paper will improve it to replace one of the random individuals $X_{A}(t)$ with the optimal individual at that time, and then add Brownian motion. The Brownian movement of the population centered on the position of the elite individuals not only enhanced the search ability of the early slime mould individuals, but also avoided rapid convergence. The formula is shown as:

(20) $$X_{b}(t)+vb\cdot \beta_{t}\cdot (W\cdot X_{b}\left(t\right)-X_{B}(t)),r<\frac{p}{2}$$

where $X_{b}\left(t\right)$ is the optimal individual, $\beta_{t}$ is Brownian motion and is also a random number with standard normal distribution, and $X_{B}(t)$ is a random individual. Then, Lévy flight strategy is added in the later$\;\frac{p}{2}$ part, that is, the stage with high food concentration. Taking advantage of Lévy’s irregular flight step length, small step length can continue to effectively conduct in-depth search in the current area, while a large step length can help the current random individuals explore the neighborhood, avoid premature convergence, and fall into local optimal. The formula is shown as:

(21) $$X_{b}\left(t\right)+vb\cdot levy(\lambda )\cdot \left(W\cdot X_{A}(t)-X_{B}\left(t\right)\right),\frac{p}{2}<r<p$$

where $X_{A}(t)$ is another random individual, and $levy\left(\;\lambda \right)\;$ is Lévy’s flight strategy. The perturbation factors α and *k* in the dandelion optimizer were added in the later iteration to further make the iteration process more diverse. To sum up, the improved position update formula is shown in Equation (22):

(22) $$X(t+1)=\left\{\begin{array}{c} X_{b}(t)+vb\cdot \beta_{t}\cdot (W\cdot X_{b}\left(t\right)-X_{B}(t)),r<\frac{p}{2}\\ X_{b}\left(t\right)+vb\cdot levy(\lambda )\cdot \left(W\cdot X_{A}(t)-X_{B}\left(t\right)\right),\frac{p}{2}<r<p\\ vc\cdot k\cdot \alpha \cdot X(t),r\geq p \end{array}\right.$$

### 3.3. Specular Reflection Learning (SRL)

**Figure 3:**
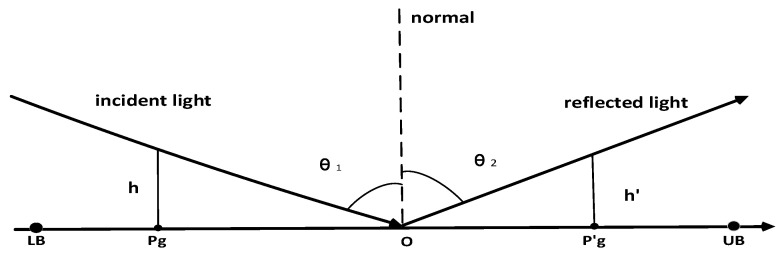
Specular reflection learning model.

Zhang proposed specular reflection learning (SRL) in 2021 [35] based on the reflection imaging law of light in flat mirrors, and the specular reflection learning model is shown in Figure 3.

In Figure 3, O is the midpoint of [LB, UB], $p_{g}$ is the optimal individual in the current population, and ${p_{g}}^{\prime }$ is the inverse individual of $p_{g}$. According to the Pythagorean theorem, we can obtain:

(23) $$\left\{\begin{array}{c} tan\theta_{1}=\frac{\left(\frac{UB+LB}{2}-p_{g}\right)}{h}\\ tan\theta_{2}=\frac{\left({p_{g}}^{\prime }-\frac{UB+LB}{2}\right)}{h^{\prime }}\; \end{array}\right.$$

Equation (24) is obtained according to $\theta_{1}$ = $\theta_{2}$:

(24) $$\frac{\left(\frac{UB+LB}{2}-p_{g}\right)}{h}\;=\;\frac{\left({p_{g}}^{\prime }-\frac{UB+LB}{2}\right)}{h^{\prime }}$$

Equation (25) presents the inverse point $p_{g}$:

(25) $${p_{g}}^{\prime }=\frac{h^{\prime }}{h}\left(\frac{UB+LB}{2}-p_{g}\right)+\frac{UB+LB}{2}$$

Let $\frac{h^{\prime }}{h}=k(k>0)$, Equation (25) can be simplified to:

(26) $${p_{g}}^{\prime }=(k+1)\cdot \frac{UB+LB}{2}-kp_{g}$$

When *k* = 1, it can be further simplified as:

(27) $${p_{g}}^{\prime }=UB+LB-p_{g}$$

Equation (27) is the general opposition-based learning applied to $p_{g}$, and it can be seen that the opposition-based learning is actually a special case of specular reflection learning. When the general opposition-based learning generalizes to the *D*-dimensional search space:

(28) $${p_{g,j}}^{\prime }=(k+1)\cdot \frac{UB_{j}+LB_{j}}{2}-kp_{g,j}$$

where *j* = 1, 2, …, *D*

Now, specular reflection learning is added to the later iteration to generate random reverse solutions, expand the diversity of the population, and avoid falling into local optimality. The calculation formula should evolve as follows:

(29) $$X\left(t+1\right)=UB+LB-{X\left(t+1\right)}^{\prime }$$

**Figure 4:**
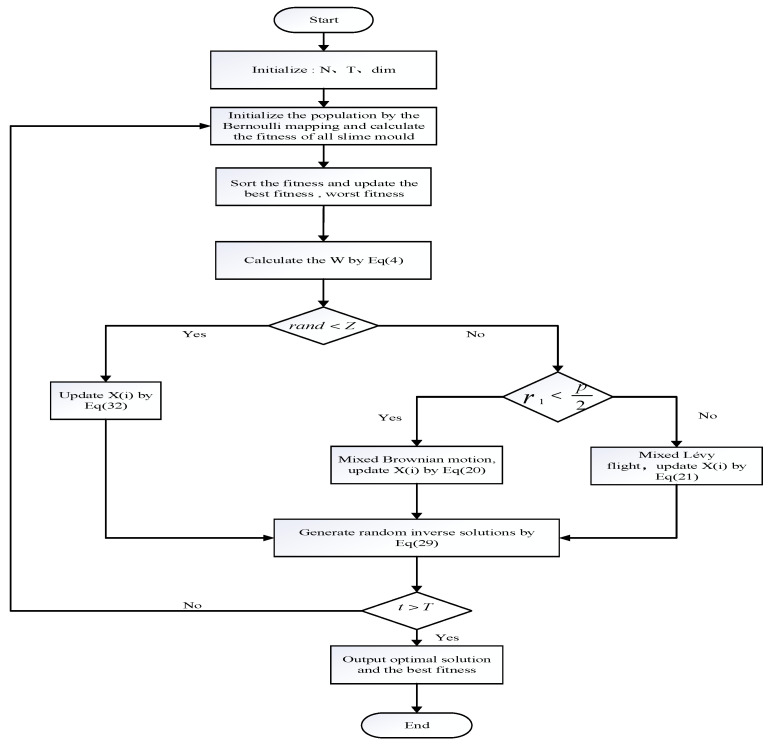
Flowchart of BDSSMA.

This paper proposed a hybrid dandelion optimizer and reflection learning method to improve the slime mould optimization algorithm (BDSSMA); its pseudocode (Algorithm 1) is as follows, and the specific process is shown in Figure 4.
**Algorithm 1.** Pseudocode of BDSSMA1: **Start**2: **Initialize** BDSSMA related parameters, such as population size N, maximum number of iterations T, variable dimension Dim, search for upper and lower bounds UB, LB.3: Generate Bernoulli map to initialize the population.4: **While** *t* < T5:  Calculate the initial fitness and select the best and worst individual.6:  Update inertia weight W according to Equation (4)7:    For *i* = 1 to N8:     **if** rand < *z*9:      Calculate the population position by Equation (32)10:    **else**
11:     **if** *r* <$\frac{p}{2}$
12:       Calculate the population position by Equation (20)13:      **if** $\frac{p}{2}$< *r* <p
14:       Calculate population location by Equation (21)15:      **end if**16:    Generate random reverse solutions by Equation (29)17:    **end** for18:    *t* = *t* + 119: **end while**20: Return the best fitness value and the best individual

## 4. Experimental Results and Analysis

This section introduces the selection experiment of the chaotic mapping function, the simulation experiment of BDSSMA under 23 standard test functions, and the comprehensive evaluation of the optimization performance of BDSSMA via the Wilcoxon rank sum test and practical engineering design problems. All experiments were run on the same operating system.

### 4.1. Experimental Environment

The simulation experiment environment is AMD Ryzen 7 5800H CPU, the main frequency is 3.20 GHz; 16 GB memory; Windows 10 (21H2) 64-bit operating system. The running software is MATLAB R2019b 64-bit.

### 4.2. Chaotic Mapping Selection

**Figure 5:**
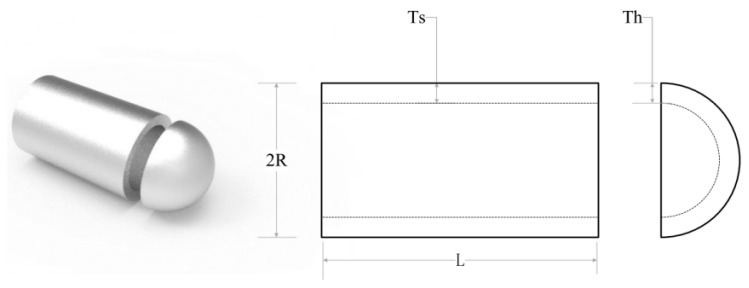
Structure of pressure vessel.

Before comparing the algorithms, the nine SMA variant algorithms are first tested to identify which chaotic mapping is better to optimize the SMA by following the method of [26] and combining it with the actual engineering problem: the pressure vessel design problem. Then, the best chaotic mapping is selected as the initialization improvement of the viscous bacteria optimization algorithm. The pressure vessel design problem is a well-known engineering design test that aims to find the parameters of a cylindrical pressure vessel that minimizes the total cost of production and meets the pressure requirements. These parameters include the thickness of the shell (Ts), the thickness of the head (Th), the inner radius (R), and the length of the cylindrical section (L). Figure 5 shows the structure of the pressure vessel.

The range of independent variables, the objective function f(x), and the four constraints g are shown below:

*X = [x*1 *x*2 *x*3 *x*4*]* = [Ts Th R L]

The objective function to deal with the problem is as follows:

(30) $$f\left(x\right)=0.6224x_{1}x_{3}x_{4}+1.7781x_{2}x_{3}^{2}+3.1661x_{1}^{2}x_{4}+19.84x_{1}^{2}x_{3}$$

Subject to the following constraints:

(31) $$\left\{\begin{array}{c} g_{1}\left(x\right)=-x_{1}+0.0193x_{3}\leq 0\\ g_{2}\left(x\right)=-x_{2}+0.00954x_{3}\leq 0\\ g_{3}\left(x\right)=-\pi x_{3}^{2}x_{4}-\frac{4}{3}\pi x_{3}^{3}+1296000\leq 0\\ g_{4}\left(x\right)=x_{4}-240\leq 0 \end{array}\right.$$

The variable ranges:

$$0\leq x_{1}\leq 99$$

$$0\leq x_{2}\leq 99$$

$$10{\leq x}_{3}\leq 200$$

$$10{\leq x}_{4}\leq 200$$

**Figure 6:**
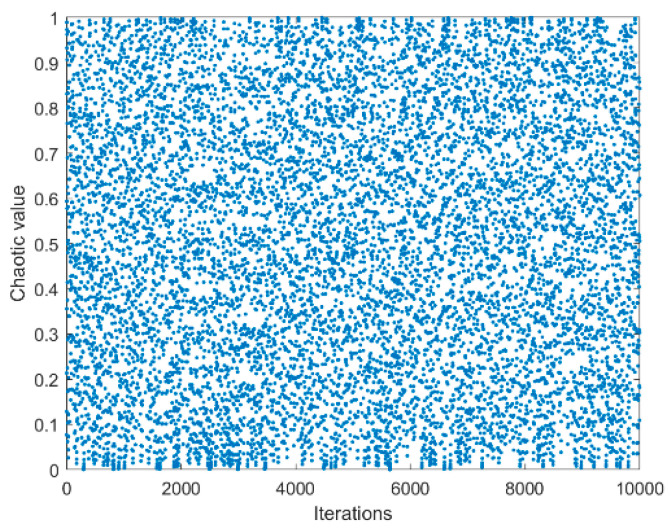
Chaos mapping scatter plot.

**Figure 7:**
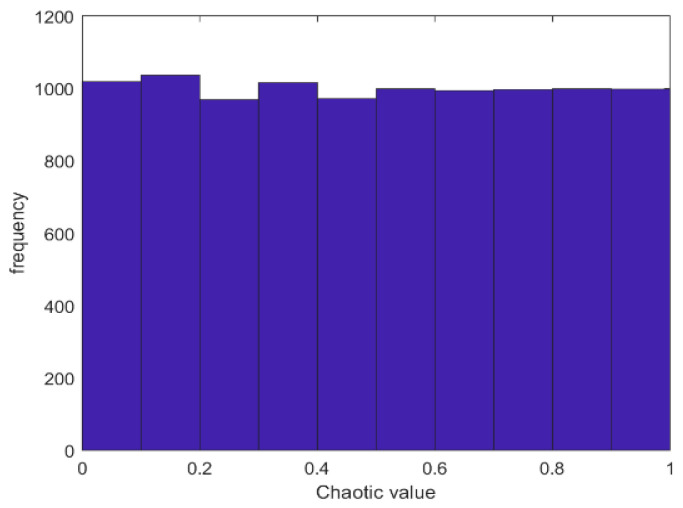
Chaos mapping histogram.

Table 2 shows the experimental data obtained by nine SMA chaotic variant algorithms when solving the pressure vessel problem. The data were averaged 30 times. As can be seen from the table, each variant algorithm can obtain better results, but the optimal value obtained by the SMA and adding Bernoulli chaotic mapping is the optimal result. Therefore, Bernoulli chaotic mapping is selected in this paper to initialize the slime mould population and expand the population diversity. The scatterplot and histogram of Bernoulli's chaotic map are shown in Figure 6 and Figure 7, and the initialization update formula of equation (32) is as follow:

Initialize the update formula:

(32) $$X\left(t+1\right)=Ber\cdot \left(UB-LB\right)+LB,\;\;rand<Z$$

### 4.3. Benchmark Function and Comparison Algorithm

The reference functions selected in this paper are 23 benchmark functions selected for algorithmic comparison. F1–F7 are unimodal functions, which have no local optimal solution and only a globally optimal solution, which is suitable for testing the algorithm’s convergence speed and global exploitation ability. F8–F13 are multi-modal functions, which have multiple local solutions in addition to the global optimal solution, and the number of local minima will increase exponentially with the increase in dimension. If the effect is not good, it quickly falls into local optimal. Therefore, this function is suitable for testing the algorithm’s ability to avoid local optimal and explore. F14–F23 are fixed-dimensional multi-modal functions, which are equivalent to the combination of the first two types of operations, with a small number of local minima, comparable to accelerated experiments, which can quickly clarify the performance of the algorithm, and are generally used to evaluate the relationship between the exploration and exploitation of algorithms. Dim represents the dimension of the function; range represents the function’s domain; and $f_{min}$ represents the optimal value of the process in Table 3.

#### 4.3.1. Test Function Experiment Results and Analysis

In order to validate the effectiveness of the hybrid modified slime mould algorithm (BDSSMA) based on dandelion optimizer proposed in this paper, we conducted a comparison amongst the slime mould algorithm (SMA) as proposed in ref. [20], the chaotic elite slime mould algorithm (CESMA) submitted in ref. [21], the marine predator algorithm (MPA) in ref. [5], the dandelion optimizer (DO) presented in ref. [1], the Sine–Cosine algorithm (SCA) [11], and the Snake Optimizer (SO) [7]—all of which were compared in terms of performance indicators such as local mining, local extreme value avoidance, and global exploration. The main parameters of each algorithm are shown in Table 4. For the fairness of comparison, all algorithms are performed under the same conditions, where the population is set to 30 and the number of iterations is set to 1000. In order to reduce the influence of random factors in the algorithms on the results, all the comparison algorithms are run in each function 30 times, respectively, and the average is taken as the final run result. The experimental results are evaluated using average value (Avg) and standard deviation (Std), and the best results are presented in bold (data requiring scientific notations are noted in three decimal places).

The BDSSMA outperforms other algorithms in most test functions in Table 5. The theoretical optimal solution is reached in F1–F5 and F7 for the unimodal test function. The convergence accuracy and stability are excellent, and F6 is better than the original SMA and the improved SMA, second only to MPA. These results show that the BDSSMA has high exploratory ability and convergence according to the characteristics of the unimodal test function. For multi-modal test functions, F8–F11 obtain the theoretical optimal value, and the MPA in F12 obtains the optimal solution. The first variance is DO, indicating that MPA and DO algorithms also have excellent global search capability, and F13 obtains the optimal solution, but the stability is slightly lower than DO. The BDSSMA can effectively avoid the local optimal solution and has an excellent global search ability according to the characteristics of the multi-modal test function. However, further improvements are stable. In the fixed dimension test function, the optimal solution of BDSSMA is obtained in F14–F23, and the convergence speed is significantly improved. However, the performance in variance could be better, which shows that although the robustness is better than the original algorithm, there are still some aspects that could be improved for MPA and SO algorithms. In summary, the BDSSMA has made significant progress in convergence accuracy and speed and has also significantly improved accuracy for MPA and DO with similar search strategies. In terms of stability, although it has made significant progress compared with the original algorithm and most algorithms, it is slightly inferior to MPA and SO. In the future, we will focus on how to balance improving accuracy and stability to make the algorithm perform better. 

**Figure 8:**
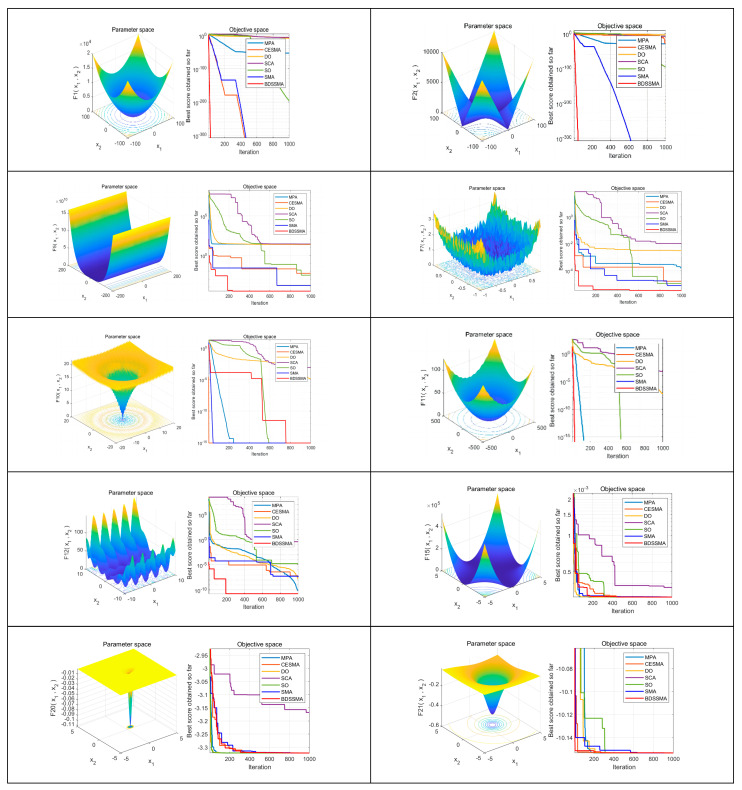
Comparison of search areas and convergence curves of some test functions.

Convergence curves for some of the test functions are shown in Figure 8. It can be seen that although other improved algorithms except the BDSSMA have high convergence accuracy, the convergence speed of F1 and F2 is too slow. It takes at least 400 iterations to reach the optimal solution. In contrast, the convergence curve of the BDSSMA decreases significantly from the beginning of the iteration. The convergence speed is breakneck; only 40 iterations are needed to reach the optimal solution. This shows that the improved search strategy dramatically enhances the searchability of slime mould in the early stage and significantly speeds up the convergence speed. It can be seen from F5 and F7 that the BDSSMA has many inflexion points, which indicates that if slime moulds fall into the local optimal prematurely in search, the possibility of slime moulds jumping out of the local optimal can be effectively improved. The improved feature of more giant steps in Lévy flight can enhance the global search ability of slime moulds. In F10, although the search enters a stagnant state in the middle period, it rapidly converges and reaches a globally optimal solution after two transitions. For F11, the curves of other algorithms may still reach the optimal solution in subsequent iterations. However, they do not converge to the optimal solution under the set number of times, which also reflects the fast convergence speed of the BDSSMA. For F12, the initial accuracy of the BDSSMA is obviously better than that of other algorithms, which reflects the improvement of chaotic mapping on the initial population, and the number of iterations and inflexion points also reflect the BDSSMA’s excellent search range and ability to jump out of the local optimal once again. Most algorithms can reach the optimal value for fixed dimension functions, F15, F19, and F21, but there are many inflexion points in the search process. At the same time, the BDSSMA has fewer inflexion points than other algorithms, further reflecting the need for improvement in global search ability. 

#### 4.3.2. Wilcoxon Rank Sum Test

Evaluating the algorithm performance via mean and standard deviation alone is not comprehensive enough, and to further evaluate the performance of BDSSMA, the Wilcoxon rank sum test nonparametric statistical test is again used here to verify whether the overall BDSSMA results have a significant advantage over the comparable algorithms. Where the significance level is set to 0.05, if the *p*-value generated by the comparison is lower than 0.05 in this case, it means that BDSSMA has a statistically significant advantage over the compared algorithm. Otherwise, the performance difference between the two algorithms could be clearer. Table 6 shows the Wilcoxon rank sum test results of BDSSMA compared with other algorithms on 23 standard test functions, where N/A indicates that the two groups of running data are identical and cannot be tested, that is, the two algorithms have the same performance. “+”, “−”, and “=“, respectively, indicate that BDSSMA is better than, worse than, and equal to the algorithm compared with it. Because the algorithm cannot be compared with itself, the *p*-value of BDSSMA is no longer listed in the table.

The BDSSMA outperforms SMA on 17 test functions, CESMA on 16 test functions, MPA on 21 test functions, DO on 21 test functions, SCA on 21 test functions, and SO on 19 test functions in Table 6. Therefore, the performance of BDSSMA is statistically significant. In conclusion, BDSSMA combines the advantages of DO and SMA and then improves the algorithm’s performance by combining the specular reflection learning strategy. The optimization accuracy and speed are higher than the other six competitive algorithms to a certain extent.

## 5. Practical Application Test of the Improved Algorithm

### 5.1. Introduction to the Principle of Extreme Learning Machine (ELM)

**Figure 9:**
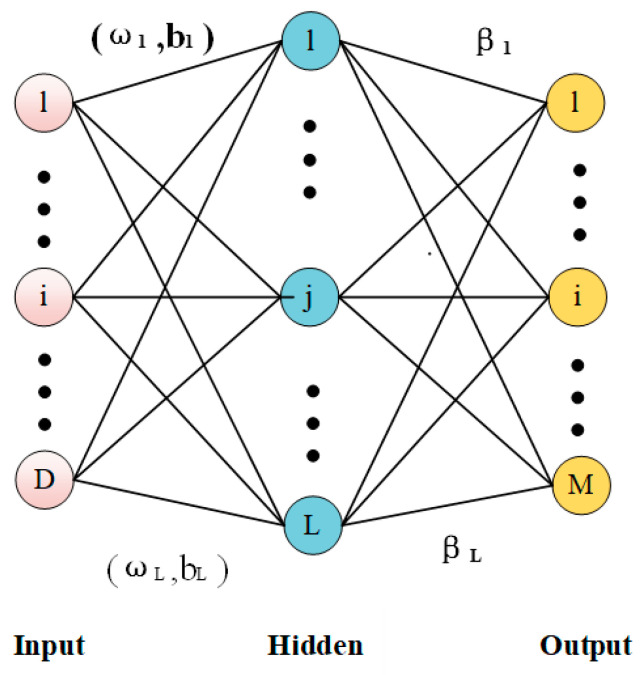
Schematic diagram of a single hidden layer neural network.

Extreme Learning Machine (ELM), proposed by Professor Huang Guangbin in 2004, is a simple, easy, and effective single-hidden layer forward neural network learning algorithm (as shown in Figure 9) [36]. Traditional neural network learning algorithms (such as the BP algorithm) must set many artificial neural network training parameters, which can easily lead to local optimal solutions. The ELM algorithm only needs to set the number of nodes in the hidden layer, does not need to adjust the input weight $\omega_{i}$ and implicit bias of the network $b_{i}$ during the implementation of the algorithm, and generates a unique optimal solution. It has the advantages of fast learning speed and good generalization performance; therefore, it has been widely used in engineering. 

The mathematical model of ELM is from Equation (33), where β denotes the matrix of output weights, H is the hidden layer output matrix, and T is the desired output matrix. After the hidden layer neuron parameters ${(\omega }_{i},b_{i})$ are randomly generated and given training samples according to arbitrary continuous sampling distribution probabilities [37], the hidden layer output matrix H is actually an invariant known quantity. Thus, Equation(34) can be obtained by solving for its minimal paradigm, where $H^{+}$ is the Moore–Penrose-generalized inverse of the minimal paradigm.

(33) Hβ=T 

(34) $$\hat{\beta }=H^{+}T.$$

### 5.2. Algorithm Performance Evaluation

The ELM algorithm can theoretically improve the learning speed of the entire network by randomly selecting hidden neuron parameters. Some studies have found that in specific circumstances [38], this feature may require ELM to have more randomly selected hidden neurons than traditional methods. Too many randomly selected neurons will inevitably produce some useless neurons for model training, failing to obtain the optimal solution. The key to optimizing ELM lies in its network structure, and meta-heuristic algorithms are gradually regarded as a new choice for optimizing ELM because of their excellent adaptive ability and search capability.

**Figure 10:**
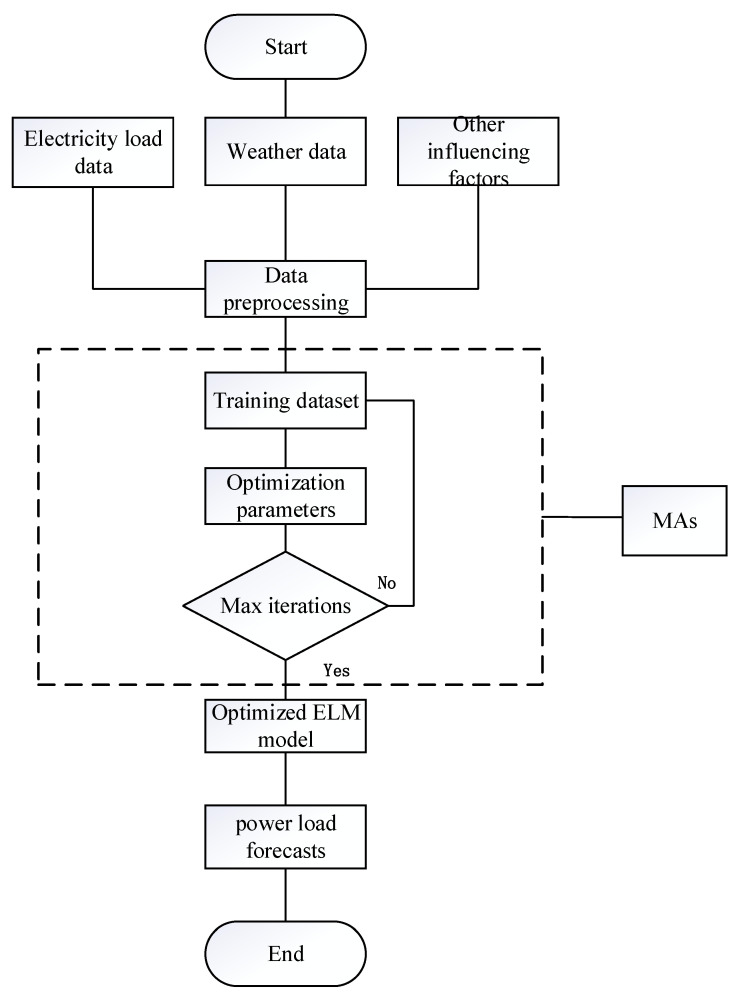
Flowchart of ELM optimized based on MAs.

This paper uses the BDSSMA to optimize ELM (refer to Figure 10). Firstly, ELM’s input weights and hidden layer biases are set to the slime individuals in the BDSSMA search space. The slime individuals continuously update their positions via the search strategy of the algorithm to update the global optimal solution. Continuous iteration searches the optimal value in the solution space to optimize the ELM model and improve the prediction results.

To validate the credibility and dependability of the proposed ELM model, this paper selected all data from the standard dataset provided by the 9th “CSEE Cup” National Electrical Mathematical Modeling Competition for College Students [39] to verify the validity and reliability of the proposed short-term load forecasting model. The dataset includes the power load value of an area from 1 January 2012 to 10 January 2015, and meteorological factor data (daily mean temperature, daily relative humidity, and daily rainfall). Data from 1 January 2014 to 14 December 2014 were used as the training set to train the ELM model, and then 96 samples were used as the test set from 15 December 2014 to 31 December 2014 to forecast the power system’s load. We will compare it with the improved ELMs of six algorithms to assess the effectiveness of BDSSMA optimized ELM in short-term load forecasting. The parameters used for the comparison will be the same as in Section 4.3.1, with 30 populations and 1000 iterations. The performance of the models will be evaluated using maximum relative error (MAE), root-mean-squared error (RMSE), and mean absolute percentage error index (MAPE), as specified in Equations (35)–(37):

(35) $$\varepsilon_{MAE}=\frac{1}{m}\sum_{i=1}^{m}\left|{p_{i}}^{\prime }-p_{i}\right|$$

(36) $$\varepsilon_{RMSE}=\sqrt{\frac{1}{m}\sum_{i=1}^{m}{{{(p}_{i}}^{\prime }-p_{i})}^{2}}$$

(37) $$\varepsilon_{MAPE}=\frac{1}{m}\sum_{i=1}^{m}\left|\frac{{p_{i}}^{\prime }-p_{i}}{p_{i}}\right|\cdot 100\%$$

where *m* is the number of samples; $p_{i}$ is the actual load value of the test set; ${p_{i}}^{\prime }$ is the predicted load value of the test set; and the smaller the$\;\varepsilon_{MAE}$, $\varepsilon_{RMSE}$, and $\varepsilon_{MAPE}$, the more accurate the prediction effect of the algorithm. 

**Figure 11:**
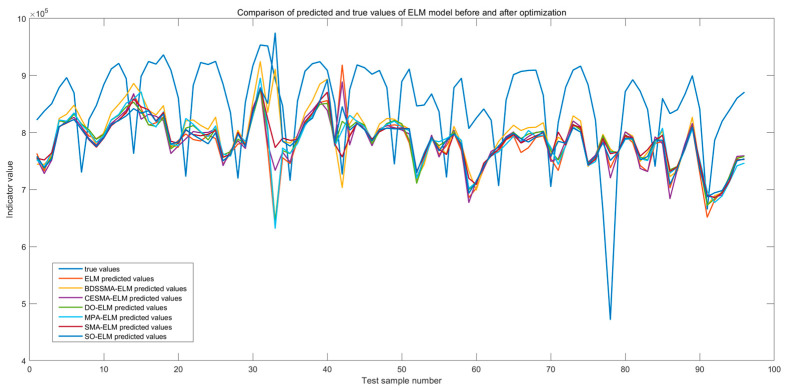
Comparison of predicted and true values of ELM model before and after optimization.

**Figure 12:**
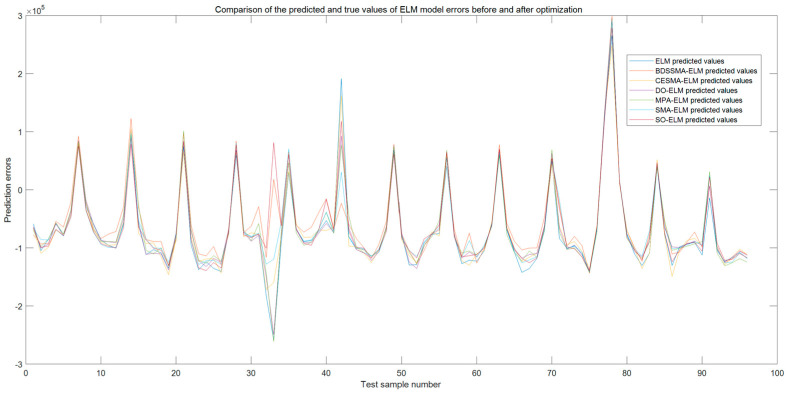
Comparison of prediction and true value errors of ELM model before and after optimization.

The changing trend of the load prediction curve of the BDSSMA-ELM model is most similar to the changing trend of the actual load. The result is closer to the real value from Figure 11 and Figure 12. However, the ELM model optimized using other classification algorithms cannot effectively predict the specific value of load and the changing trend. Table 7 shows the accuracy of different ELM prediction models to compare the prediction accuracy of different models more directly.

The maximum relative error $\varepsilon_{MAE}$ of the BDSSMA-ELM decreased by 14.053 KW, 8.535 KW, 6.886 KW, 10.167 KW, 11.130 KW, and 10.167 KW, respectively, compared with traditional ELM, SMA-ELM, CESMA-ELM, MPA-ELM, DO-ELM, and SO-ELM prediction models. The root-mean-squared error $\varepsilon_{MAPE}$ decreased by 13.691 KW, 5.854 KW, 4.697 KW, 9.207 KW, 8.717 KW, and 9.207 KW, respectively. The mean absolute percentage error $\varepsilon_{MAPE}$ decreased by 1.6469%, 1.0353%, 0.8337%, 1.2259%, 1.3073%, and 1.2259%, respectively. In summary, The ELM combined with BDSSMA has made some progress in prediction accuracy. There are still two main problems to be solved. First, the improved algorithm shows good application results on the test set. However, the load will have a certain number of discrete points in the graph with large fluctuations in the actual power operation problem. It will significantly impact the prediction effect of ELM. There is still a gap with the actual value, although this error can be reduced by adding an optimization algorithm. How to optimize the data preprocessing is a problem in the future. Secondly, the algorithm’s running time is longer, although the improved algorithm improves the ELM prediction accuracy and enhances the stability. Our next focus is how to strike a balance between improving efficiency and reducing time.

## 6. Conclusions

This paper presents an improved BDSSMA by referring to the dandelion optimizer and some strategies to optimize the slime mould algorithm in the exploration and exploitation stages. (1) Different variants are selected in the initialization stage. The Bernoulli chaos map is finally selected to increase the population diversity. (2) It is proposed to divide the molds’ position update variable p into two stages and then mix the excellent mechanism of stage exploration and exploitation in the dandelion optimizer, which has exceptional global search ability so that the algorithm adopts different position update formulas at different stages to enhance further the global search ability and local exploitation performance of moulds. (3) The specular reflection learning strategy was introduced in the late iteration to help further the slime mould population escape from local optimization and improve the solution accuracy. A series of standard test function experiments show that the proposed improved algorithm performs better than SMA, CESMA, MPA, DO, SCA, and SO in convergence speed and accuracy. In the Wilcoxon rank sum test, BDSSMA also achieved excellent results in a statistical sense. In the actual ELM model of power load forecasting, the forecasting accuracy has also been improved. In this paper, the accuracy and speed of the SMA global optimization are improved under ideal simulation test conditions. However, the improved algorithm still has many limitations when faced with practical engineering problems with many constraints. How to improve the stability of the improved algorithm and how the improved algorithm can further optimize the prediction time are the directions that should be focused on in future research.

## Figures and Tables

**Table 1:** Chaotic mapping set.

Variant Name	Chaotic Map Strategy	Range
Chebyshev	$X_{n+1}=\cos\left(n{cos}^{-1}(X_{n}\right))$	[−1, 1]
Improved Chebyshev	$X_{n+1}=1-2{\left(\cos\left(2arccosX_{n}\right)\right)}^{2}$	[−1, 1]
SPM	$X_{n+1}\left\{\begin{array}{c} mod\frac{X_{n}}{\gamma }+\mu \sin\left(\pi X_{n}\right)+r,0<X_{n}<\gamma \\ mod\frac{X_{n}}{\gamma (0.5-\gamma )}+\mu \sin\left(\pi X_{n}\right)+r,\gamma <X_{n}<0.5\\ F\left(1-X_{n},\gamma ,\mu \right),0.5<X_{n}<1 \end{array}\right.$	(0, 1)
Neuron	$X_{n+1}=\delta -2tanh\left(\gamma \right)exp\left(-3{X_{n}}^{2}\right),0<\delta <1$	(0, 1)
Bernoulli	$X_{n+1}=\left\{\begin{array}{c} \frac{X_{n}}{1-\gamma },{0<X}_{n}\leq 1-\gamma \\ \frac{X_{n}-(1-\gamma )}{\gamma },{1-\gamma <X}_{n}<1 \end{array}\right.$	(0, 1)
Henon	$\left\{\begin{array}{c} X_{n+1}=1+Y_{n}-aX_{n}^{2}\\ Y_{n+1}=bX_{n} \end{array}\right.$	(0, 1)
Kent	$X_{n+1}=\left\{\begin{array}{c} \frac{X_{n}}{m},{0<X}_{n}\leq m\\ \frac{1-X_{n}}{1-m},{m<X}_{n}<1 \end{array}\right.$	(0, 1)
Fuch	$X_{n+1}=cos(\frac{1}{{X_{n}}^{2}}$)	(0, 1)
Preferred point set	$X_{n+1}=2\cos\left(\frac{2\pi x}{t}\right),t\geq 2s+3$	(1, s)

**Table 2:** Comparison of the results of pressure vessel design problems with other chaos variants.

Variant Name	Ts	Th	R	L	Optimal Cost
**SMA**	0.8581984	0.4254994	44.4648	149.368	6050.9863
**ChebyshevSMA**	0.8135765	0.4025301	42.15186	175.9837	5950.3339
**Improved ChebyshevSMA**	0.7792018	0.38527	40.37239	199.2677	5887.5434
**SPMSMA**	0.8162131	0.4035017	42.29017	174.2755	5953.8328
**NeuronSMA**	0.8268668	0.4087785	42.84112	167.6462	5974.2974
**BernoulliSMA**	**0.7782284**	**0.3850124**	**40.32229**	**199.9883**	**5887.0029**
**HenonSMA**	0.7931507	0.3920965	41.09537	189.479	5911.7051
**KentSMA**	0.8639112	0.4273306	44.76207	146.2083	6049.7537
**FuchSMA**	0.7989452	0.3951562	41.39605	185.5404	5922.5633
**GoodsetSMA**	0.8309936	0.4107689	43.05643	165.1172	5981.9675

**Table 3:** Standard test functions.

Functions	Dim	Range	$f_{min}$
$F_{1}\left(x\right)=\sum_{i=1}^{n}x_{i}^{2}$	30	[−100, 100]	0
$F_{2}\left(x\right)=\sum_{i=1}\left|x_{i}\right|+\prod_{i=1}^{n}\left|x_{i}\right|$	30	[−10, 10]	0
$F_{3}\left(x\right)=\sum_{i=1}{(\sum_{j-1}^{i}x_{j})}^{2}$	30	[−100, 100]	0
$F_{4}\left(x\right)=max_{i}\{\left|x_{i}\right|,1\leq i\leq n\}$	30	[−100, 100]	0
$F_{5}\left(x\right)=\sum_{i=1}^{n-1}\left(100{\left(x_{i}^{2}-x_{i+1}\right)}^{2}+{\left(x_{i}-1\right)}^{2}\right)$	30	[−30, 30]	0
$F_{6}\left(x\right)=\sum_{i=1}^{n}{\left(x_{i}+0.5\right)}^{2}$	30	[−100, 100]	0
$F_{7}\left(x\right)=\sum_{i=1}^{n}ix_{i}^{4}+random\;[0,\;1]$	30	[−128, 128]	0
$F_{8}\left(x\right)=\sum_{i=1}^{n}-x_{i}sin(\sqrt{\left|x_{i}\right|})$	30	[−500, 500]	−2094.9145
$F_{9}\left(x\right)=\sum_{i=1}^{n}\left(x_{i}^{2}-10cos\left(2\pi x_{i}\right)+10\right)$	30	[−5.12, 5.12]	0
$F_{10}\left(x\right)=-20exp\left(-0.2\sqrt{\frac{1}{n}}\sum_{i=1}^{n}x_{i}^{2}\right)-exp\left(\frac{1}{n}\sum_{i=1}^{n}cos\left(2\pi x_{i}\right)\right)$+20+e	30	[−32, 32]	0
$F_{11}\left(x\right)=\frac{1}{4000}\sum_{i=1}^{n}x_{i}^{2}-\prod_{i=1}^{n}\cos\left(\frac{x_{i}}{\sqrt{i}}\right)+1$	30	[−600, 600]	0
$F_{12}\left(x\right)=\frac{\pi }{n}\left\{10\sin\left(\pi y_{1}\right)+\sum_{i=1}^{n-1}{\left(y_{i}-1\right)}^{2}\left[1+10{sin}^{2}\left(\pi y_{i+1}\right)\right]+{\left(y_{n}-1\right)}^{2}\right\}+\sum_{i=1}^{n}u\left(x_{i},10,100,4\right)$ $y_{i}=1+\frac{x_{i}+1}{4}$ u$\left(x_{i},a,k,m\right)=\left\{\begin{array}{c} k{\left(x_{i}-a\right)}^{m}\;\;\;\;\;\;\;\;\;\;\;\;\;\;\;\;\;\;\;\;\;\;\;\;\;\;\;\;\;\;\;\;\;\;\;\;x_{i}>a\\ 0\;\;\;\;\;\;\;\;\;\;\;\;\;\;\;\;\;\;\;\;\;\;\;\;\;\;\;\;\;\;\;\;\;\;\;\;\;\;\;\;-a<x_{i}<a\\ k{\left(-x_{i}-a\right)}^{m}\;\;\;\;\;\;\;\;\;\;\;\;\;\;\;\;\;\;\;\;\;\;\;\;\;\;\;x_{i}<-a\;\;\; \end{array}\right.$	30	[−50, 50]	0
$F_{13}\left(x\right)=0.1\left\{{sin}^{2}\left(3\pi x_{1}\right)+\sum_{i=1}^{n}{\left(x_{i}-1\right)}^{2}\left[1+{sin}^{2}(3\pi x_{i}+1)\right]+{\left(x_{n}-1\right)}^{2}\left[1+10{sin}^{2}\left(2\pi x_{n}\right)\right]\right\}+\sum_{i=1}^{n}u(x_{i},5,100,4)$	30	[−50, 50]	0
$F_{14}\left(x\right)={\left(\frac{1}{500}+\sum_{j=1}^{25}\frac{1}{j+\sum_{i=2}^{2}{\left(x_{i}-a_{ij}\right)}^{6}}\right)}^{-1}$	2	[−65.536, −65.536]	1
$F_{15}\left(x\right)=\sum_{i=1}^{11}{\left[a_{i}-\frac{x_{1}(b_{i}^{2}+b_{i}x_{2}}{b_{i}^{2}+b_{i}x_{3}+x_{4}}\right]}^{2}$	4	[−5, 5]	0.0003
$F_{16}\left(x\right)=4{x_{1}}^{2}-2.1{x_{1}}^{4}+\frac{1}{3}{x_{1}}^{6}+x_{1}x_{2}-4{x_{2}}^{2}+4{x_{2}}^{4}$	2	[−5, 5]	−1.0316
$F_{17}\left(x\right)={\left(x_{2}-\frac{5.1}{4\pi^{2}}{x_{1}}^{2}+\frac{5}{\pi }x_{1}-6\right)}^{2}+10\left(1-\frac{1}{8\pi }\right)cosx_{1}+10$	2	[−5, 5]	0.398
$F_{18}\left(x\right)=\left[1+{\left(x_{1}+x_{2}+1\right)}^{2}\left(19-14x_{1}+3{x_{1}}^{2}-14x_{2}+6x_{1}x_{2}+3{x_{2}}^{2}\right)\right]\times [30+{\left(2x_{1}-3x_{2}\right)}^{2}\times (18-32x_{1}+12{x_{1}}^{2}-48x_{2}+36x_{1}x_{2}+27{x_{2}}^{2})]$	2	[−2, 2]	3
$F_{19}\left(x\right)=-\sum_{i=1}^{4}c_{i}exp\left[-\sum_{j=1}^{3}a_{ij}{\left(x_{j}-p_{ij}\right)}^{2}\right]$	3	[1, 3]	−3.86
$F_{20}\left(x\right)=-\sum_{i=1}^{4}c_{i}exp\left[-\sum_{j=1}^{6}a_{ij}{\left(x_{j}-p_{ij}\right)}^{2}\right]$	6	[0, 1]	−3.32
$F_{21}\left(x\right)=-\sum_{i=1}^{5}{\left[\left(X-a_{i}\right){\left(X-a_{i}\right)}^{T}+c_{i}\right]}^{-1}$	4	[0, 10]	−10.5363
$F_{22}\left(x\right)=-\sum_{i=1}^{7}{\left[\left(X-a_{i}\right){\left(X-a_{i}\right)}^{T}+c_{i}\right]}^{-1}$	4	[0, 10]	−10.4028
$F_{23}\left(x\right)=-\sum_{i=1}^{10}{\left[\left(X-a_{i}\right){\left(X-a_{i}\right)}^{T}+c_{i}\right]}^{-1}$	4	[0, 10]	−10.5363

**Table 4:** Parameter settings of the competitors.

Algorithms	Parameters
SMA	Z = 0.03, N = 30
CEMSA	Z = 0.03, N = 30
MPA	FADs = 0.2, P = 0.5
DO	α = [0, 1], k = [0, 1], N = 30
SCA	a = 2, r1 = r2, r4 = [0, 1]
SO	N = 30, T = 0.25, T1 = 0.6 C1 = 0.5, C2 = 0.05, C3 = 2

**Table 5:** Test functions’ optimization results of different algorithms.

Functions		BDSSMA	SMA	CESMA	MPA	DO	SCA	SO
**F1**	**Avg**	0	0	0	$3.242\times 10^{-50}$	$3.043\times 10^{-7}$	$3.565\times 10^{-1}$	$6.127\times 10^{-190}$
	**Std**	0	0	0	$5.078\times 10^{-50}$	$2.052\times 10^{-7}$	1.8701	$6.598\times 10^{-190}$
**F2**	**Avg**	0	0	$3.188\times 10^{-121}$	$5.632\times 10^{-28}$	$4.179\times 10^{-4}$	$9.579\times 10^{-5}$	$2.840\times 10^{-93}$
	**Std**	0	0	$9.844\times 10^{-121}$	$1.491\times 10^{-27}$	$2.107\times 10^{-4}$	$2.037\times 10^{-4}$	$7.865\times 10^{-93}$
**F3**	**Avg**	0	0	$5.760\times 10^{-181}$	$1.018\times 10^{-10}$	2.9564	5793.5845	$1.369\times 10^{-117}$
	**Std**	0	0	0	$4.993\times 10^{-10}$	2.4232	3059.3679	$4.811\times 10^{-117}$
**F4**	**Avg**	0	0	$3.313\times 10^{-113}$	$2.567\times 10^{-19}$	$2.737\times 10^{-1}$	28.0972	$6.320\times 10^{-84}$
	**Std**	0	0	$1.506\times 10^{-112}$	$2.193\times 10^{-19}$	$1.914\times 10^{-1}$	12.8361	$1.434\times 10^{-83}$
**F5**	**Avg**	$7.805\times 10^{-2}$	5.4668	$4.738\times 10^{-1}$	24.3038	27.3367	5750.7596	15.4106
	**Std**	$1.655\times 10^{-1}$	10.4531	$6.969\times 10^{-1}$	$3.679\times 10^{-1}$	3.8289	13179.8133	12.4418
**F6**	**Avg**	$5.851\times 10^{-4}$	$6.754\times 10^{-4}$	$7.341\times 10^{-4}$	$1.914\times 10^{-9}$	$9.748\times 10^{-7}$	4.6753	$1.115\times 10^{-1}$
	**Std**	$1.598\times 10^{-4}$	$3.152\times 10^{-4}$	$3.772\times 10^{-4}$	$8.705\times 10^{-10}$	$3.850\times 10^{-7}$	5.1547	1.4621
**F7**	**Avg**	$9.719\times 10^{-5}$	$1.181\times 10^{-4}$	$9.276\times 10^{-4}$	$7.491\times 10^{-4}$	$1.233\times 10^{-2}$	$4.192\times 10^{-2}$	$1.629\times 10^{-4}$
	**Std**	$9.795\times 10^{-5}$	$8.321\times 10^{-5}$	$7.673\times 10^{-4}$	$3.527\times 10^{-4}$	$5.401\times 10^{-3}$	$3.403\times 10^{-2}$	$1.275\times 10^{-4}$
**F8**	**Avg**	−12569.4586	−12569.4083	−12567.3592	−9732.7816	−8365.2092	−4005.6199	−12548.3644
	**Std**	$3.553\times 10^{-2}$	$5.525\times 10^{-2}$	$4.250\times 10^{-2}$	437.6714	825.8337	282.9489	36.9470
**F9**	**Avg**	0	0	0	0	17.9632	16.1379	$5.106\times 10^{-1}$
	**Std**	0	0	0	0	19.9646	21.3463	$1.089\times 10^{-3}$
**F10**	**Avg**	$8.882\times 10^{-16}$	$8.882\times 10^{-16}$	$8.882\times 10^{-16}$	$4.086\times 10^{-15}$	$2.869\times 10^{-5}$	11.4109	$1.346\times 10^{-15}$
	**Std**	0	0	0	$1.388\times 10^{-15}$	$1.903\times 10^{-6}$	10.0663	0
**F11**	**Avg**	0	0	0	0	$-6.641\times 10^{-4}$	$-7.558\times 10^{-1}$	$6.136\times 10^{-3}$
	**Std**	0	0	0	0	$4.596\times 10^{-4}$	$2.616\times 10^{-1}$	$1.025\times 10^{-2}$
**F12**	**Avg**	$2.209\times 10^{-5}$	$2.265\times 10^{-4}$	$2.916\times 10^{-4}$	$6.301\times 10^{-7}$	$6.785\times 10^{-7}$	2.3779	$3.803\times 10^{-2}$
	**Std**	$1.362\times 10^{-5}$	$8.394\times 10^{-3}$	$8.542\times 10^{-4}$	$8.560\times 10^{-8}$	$2.318\times 10^{-8}$	3.8311	$6.831\times 10^{-2}$
**F13**	**Avg**	$1.542\times 10^{-4}$	$4.339\times 10^{-3}$	$1.941\times 10^{-3}$	$3.453\times 10^{-3}$	$9.762\times 10^{-4}$	206.1985	$9.149\times 10^{-3}$
	**Std**	$8.185\times 10^{-4}$	$5.619\times 10^{-3}$	$2.861\times 10^{-3}$	$1.422\times 10^{-2}$	$3.449\times 10^{-7}$	918.8782	$1.236\times 10^{-2}$
**F14**	**Avg**	−1.0316	−1.0316	−1.0316	−1.0316	−1.0316	−1.0316	−1.0316
	**Std**	$2.952\times 10^{-13}$	$4.172\times 10^{-10}$	$5.462\times 10^{-11}$	$6.663\times 10^{-16}$	$8.506\times 10^{-14}$	$9.247\times 10^{-5}$	$2.561\times 10^{-16}$
**F15**	**Avg**	$2.944\times 10^{-4}$	$5.318\times 10^{-4}$	$4.009\times 10^{-4}$	$3.075\times 10^{-4}$	$9.645\times 10^{-4}$	$1.016\times 10^{-3}$	$4.282\times 10^{-4}$
	**Std**	$2.846\times 10^{-4}$	$3.575\times 10^{-4}$	$2.427\times 10^{-4}$	$1.485\times 10^{-19}$	$4.105\times 10^{-4}$	$2.041\times 10^{-4}$	$3.575\times 10^{-4}$
**F16**	**Avg**	0.998	0.998	0.998	0.998	0.998	1.3954	0.99911
	**Std**	$6.422\times 10^{-17}$	$1.443\times 10^{-13}$	$5.922\times 10^{-14}$	$3.559\times 10^{-15}$	$6.077\times 10^{-16}$	$2.433\times 10^{-3}$	$6.736\times 10^{-3}$
**F17**	**Avg**	$3.979\times 10^{-1}$	$3.979\times 10^{-1}$	$3.979\times 10^{-1}$	$3.979\times 10^{-1}$	$3.979\times 10^{-1}$	0.3987	$3.979\times 10^{-1}$
	**Std**	$1.766\times 10^{-9}$	$3.502\times 10^{-9}$	$2.071\times 10^{-9}$	$6.773\times 10^{-12}$	$1.650\times 10^{-12}$	$1.731\times 10^{-4}$	$6.409\times 10^{-14}$
**F18**	**Avg**	3	3	3	3	3	3	6.227
	**Std**	$2.876\times 10^{-15}$	$2.172\times 10^{-12}$	$1.321\times 10^{-12}$	$7.675\times 10^{-15}$	$3.103\times 10^{-9}$	$3.869\times 10^{-5}$	9.043
**F19**	**Avg**	−3.8628	−3.8628	−3.8628	−3.8628	−3.8628	−3.8552	−3.8628
	**Std**	$4.286\times 10^{-10}$	$3.371\times 10^{-8}$	$2.106\times 10^{-7}$	$2.710\times 10^{-15}$	$1.475\times 10^{-8}$	$2.596\times 10^{-4}$	$2.954\times 10^{-15}$
**F20**	**Avg**	−3.3446	−3.2386	−3.2356	−3.3220	−3.2655	−2.8703	−3.3131
	**Std**	$5.750\times 10^{-2}$	$5.597\times 10^{-2}$	$5.388\times 10^{-2}$	$1.488\times 10^{-15}$	$6.013\times 10^{-2}$	$3.916\times 10^{-1}$	$1.714\times 10^{-2}$
**F21**	**Avg**	−10.1532	−10.1531	−10.1530	−10.1532	−5.3945	−3.158	−10.1496
	**Std**	$8.389\times 10^{-5}$	$7.476\times 10^{-5}$	$4.428\times 10^{-4}$	$5.891\times 10^{-15}$	3.3004	2.0671	$8.222\times 10^{-3}$
**F22**	**Avg**	−10.4028	−10.4027	−10.4019	−10.4029	−6.7583	−3.1367	−10.3981
	**Std**	$4.778\times 10^{-4}$	$1.141\times 10^{-4}$	$2.572\times 10^{-4}$	$3.506\times 10^{-15}$	3.178	1.7267	$1.037\times 10^{-2}$
**F23**	**Avg**	−10.5363	−10.5363	−10.5219	−10.5364	−7.0434	−4.6063	−10.5364
	**Std**	$9.941\times 10^{-5}$	$7.145\times 10^{-5}$	$2.391\times 10^{-2}$	$3.181\times 10^{-15}$	3.8456	1.587	0.0099343

**Table 6:** Wilcoxon rank sum test results.

Functions	SMA	CESMA	MPA	DO	SCA	SO
**F1**	N/A	N/A	$1.212\times 10^{-12}$	$1.212\times 10^{-12}$	$1.212\times 10^{-12}$	$1.212\times 10^{-12}$
**F2**	N/A	N/A	$8.007\times 10^{-9}$	$8.007\times 10^{-9}$	$8.007\times 10^{-9}$	$8.007\times 10^{-9}$
**F3**	N/A	$2.934\times 10^{-2}$	$1.212\times 10^{-12}$	$1.212\times 10^{-12}$	$1.212\times 10^{-12}$	$1.212\times 10^{-12}$
**F4**	N/A	$4.193\times 10^{-2}$	$1.212\times 10^{-12}$	$1.212\times 10^{-12}$	$1.212\times 10^{-12}$	$1.212\times 10^{-12}$
**F5**	$7.695\times 10^{-8}$	$1.800\times 10^{-8}$	$3.019\times 10^{-11}$	$3.019\times 10^{-11}$	$3.019\times 10^{-11}$	$8.485\times 10^{-9}$
**F6**	$3.439\times 10^{-2}$	$5.592\times 10^{-1}$	$3.019\times 10^{-11}$	$3.019\times 10^{-11}$	$3.019\times 10^{-11}$	$3.019\times 10^{-11}$
**F7**	$3.095\times 10^{-2}$	$4.127\times 10^{-2}$	$1.329\times 10^{-10}$	$3.019\times 10^{-11}$	$3.019\times 10^{-11}$	$9.524\times 10^{-3}$
**F8**	$1.892\times 10^{-4}$	$2.745\times 10^{-3}$	$3.019\times 10^{-11}$	$3.019\times 10^{-11}$	$3.019\times 10^{-11}$	$1.206\times 10^{-10}$
**F9**	N/A	N/A	N/A	$1.212\times 10^{-12}$	$1.212\times 10^{-12}$	$8.152\times 10^{-2}$
**F10**	$8.818\times 10^{-3}$	N/A	$5.359\times 10^{-5}$	$6.386\times 10^{-5}$	$6.386\times 10^{-5}$	$1.594\times 10^{-5}$
**F11**	N/A	N/A	N/A	$6.386\times 10^{-5}$	$6.386\times 10^{-5}$	$2.004\times 10^{-2}$
**F12**	$6.553\times 10^{-11}$	$3.604\times 10^{-7}$	$6.796\times 10^{-13}$	$1.201\times 10^{-6}$	$6.796\times 10^{-14}$	$5.896\times 10^{-14}$
**F13**	$3.771\times 10^{-9}$	$4.084\times 10^{-5}$	$8.292\times 10^{-5}$	$1.957\times 10^{-9}$	$3.019\times 10^{-14}$	$1.235\times 10^{-1}$
**F14**	$6.796\times 10^{-3}$	$1.465\times 10^{-3}$	$2.717\times 10^{-19}$	$5.008\times 10^{-17}$	$7.066\times 10^{-18}$	$1.913\times 10^{-18}$
**F15**	$3.018\times 10^{-2}$	$3.012\times 10^{-2}$	$3.010\times 10^{-14}$	$2.281\times 10^{-2}$	$6.526\times 10^{-9}$	$6.588\times 10^{-2}$
**F16**	$6.796\times 10^{-3}$	$1.396\times 10^{-1}$	$6.796\times 10^{-14}$	$6.777\times 10^{-14}$	$7.899\times 10^{-14}$	$2.205\times 10^{-2}$
**F17**	$7.389\times 10^{-3}$	$1.199\times 10^{-2}$	$1.777\times 10^{-10}$	$6.627\times 10^{-14}$	$3.019\times 10^{-14}$	$2.374\times 10^{-13}$
**F18**	$1.022\times 10^{-5}$	$1.468\times 10^{-4}$	$8.172\times 10^{-15}$	$1.429\times 10^{-14}$	$7.319\times 10^{-14}$	$9.323\times 10^{-9}$
**F19**	$7.727\times 10^{-3}$	$2.709\times 10^{-2}$	$1.212\times 10^{-12}$	$6.736\times 10^{-6}$	$3.019\times 10^{-11}$	$4.081\times 10^{-12}$
**F20**	$2.320\times 10^{-2}$	$4.911\times 10^{-2}$	$1.475\times 10^{-15}$	$3.857\times 10^{-7}$	$1.435\times 10^{-14}$	$2.196\times 10^{-13}$
**F21**	$8.684\times 10^{-4}$	$6.121\times 10^{-3}$	$7.574\times 10^{-12}$	$7.959\times 10^{-3}$	$3.019\times 10^{-11}$	$6.621\times 10^{-1}$
**F22**	$9.748\times 10^{-6}$	$3.705\times 10^{-5}$	$1.512\times 10^{-8}$	$2.853\times 10^{-1}$	$6.796\times 10^{-8}$	$5.991\times 10^{-3}$
**F23**	$3.183\times 10^{-3}$	$7.380\times 10^{-6}$	$1.015\times 10^{-11}$	$3.672\times 10^{-1}$	$3.019\times 10^{-11}$	$1.688\times 10^{-3}$
+/-/=	17/0/6	16/2/5	21/0/2	21/2/0	23/0/0	19/4/0

**Table 7:** Comparison of the accuracy of different ELM prediction models.

Prediction Model	$\varepsilon_{MAE}$/KW	$\varepsilon_{RMSE}$/KW	$\varepsilon_{MAPE}$/%
ELM	95.701	103.875	11.4127%
BDSSMA-ELM	**81.648**	**90.184**	**9.7658%**
SMA-ELM	90.183	96.038	10.8011%
CESMA-ELM	88.534	94.881	10.5995%
MPA-ELM	91.815	99.391	10.9917%
DO-ELM	92.778	98.901	11.0731%
SO-ELM	91.815	99.391	10.9917%

## Data Availability

The original contributions presented in the study are included in the article, further inquiries can be directed to the corresponding author.
